# Membrane pyrophosphatases from *Thermotoga maritima* and *Vigna radiata* suggest a conserved coupling mechanism

**DOI:** 10.1038/ncomms13596

**Published:** 2016-12-06

**Authors:** Kun-Mou Li, Craig Wilkinson, Juho Kellosalo, Jia-Yin Tsai, Tommi Kajander, Lars J. C. Jeuken, Yuh-Ju Sun, Adrian Goldman

**Affiliations:** 1 Department of Life Science and Institute of Bioinformatics and Structural Biology, College of Life Science, National Tsing Hua University, Hsinchu 30013, Taiwan; 2 Astbury Centre for Structural Molecular Biology, School of Biomedical Sciences, University of Leeds, Leeds LS2 9JT, England; 3 Division of Biochemistry, Department of Biosciences, University of Helsinki, Helsinki, FIN-00014, Finland; 4 Institute of Biotechnology, University of Helsinki, PO Box 65, Helsinki, FIN-00014, Finland

## Abstract

Membrane-bound pyrophosphatases (M-PPases), which couple proton/sodium ion transport to pyrophosphate synthesis/hydrolysis, are important in abiotic stress resistance and in the infectivity of protozoan parasites. Here, three M-PPase structures in different catalytic states show that closure of the substrate-binding pocket by helices 5–6 affects helix 13 in the dimer interface and causes helix 12 to move down. This springs a ‘molecular mousetrap', repositioning a conserved aspartate and activating the nucleophilic water. Corkscrew motion at helices 6 and 16 rearranges the key ionic gate residues and leads to ion pumping. The pumped ion is above the ion gate in one of the ion-bound structures, but below it in the other. Electrometric measurements show a single-turnover event with a non-hydrolysable inhibitor, supporting our model that ion pumping precedes hydrolysis. We propose a complete catalytic cycle for both proton and sodium-pumping M-PPases, and one that also explains the basis for ion specificity.

## Abstract (web summary)

In some parasites, membrane-bound pyrophosphatases, which couple proton and sodium ion transport across the membrane, are important for infectivity. Here, the authors report crystal structures of these proteins alongside biophysical analyses that allow them to propose a model for how the coupling is achieved.

Membrane-bound pyrophosphatases (M-PPases) are enzymes that couple the synthesis or hydrolysis of pyrophosphate (PP_i_) to the vectorial transport of protons and/or sodium ions^1^. These enzymes are found in plants and in various unicellular organisms and are important for survival under different abiotic stress conditions, such as drought and cold stress in plants^2^. The stress resistance conveyed by M-PPases is presumably due to the capability of the enzymes to use the metabolic by-product, PP_i_, for creating transmembrane cation potential differences that can drive various cellular processes, including ATP synthesis and secondary transport^3^.

M-PPases utilize Mg_2_PP_i_ as their substrate and require Mg^2+^ for activity, binding two activating Mg^2+^ (*K*_d_s 20–42 μM and 0.25–0.46 mM)^2^ with an additional inhibitory Mg^2+^ binding site (*K*_d_≈100 mM)^4^ present in the active site. In addition, M-PPases can be divided into seven functional classes on the basis of their monovalent cation binding (K^+^-dependent and K^+^-independent enzymes) and pumping specificity (H^+^-pumps (H^+^-PPases), Na^+^-pumps (Na^+^-PPases) and H^+^ and Na^+^ co-pumps (Na^+^/H^+^-PPases)) and on the placement of a ‘semi-conserved glutamate' essential for pumping activity^3^,^4^,^5^. K^+^-dependent enzymes with a conserved alanine ‘*A*^12.46^' (*Thermotoga maritima* Na^+^-PPase (TmPPase) A495 and *Vigna radiata* H^+^-PPase (VrPPase) A537) are activated by monovalent cations, of which K^+^ shows the highest level of activation^2^. K^+^-independent enzymes, on the other hand, contain a conserved lysine ‘*K*^12.46^' where the Nζ amino group probably substitutes for the bound cation of the K^+^-dependent enzymes^6^. (In what follows, we refer to residues as in refs 3, 4, 5, 7, represented as: X^Y.Z^ where X is the amino acid, *Y* is the helix number and Z is the residue position based on a conserved residue found within the helices of all known M-PPases: Supplementary Table 1.)

The semi-conserved glutamate is in the same position (*E*^6.53^) in the K^+^-independent H^+^-PPases as well as the (K^+^-dependent) Na^+^-PPases and Na^+^/H^+^-PPases. This position differs in the K^+^-dependent H^+^-PPases (*E*^5.43^, *E*^6.53^ or *E*^6.57^)^1^,^8^.

Both Na^+^-PPases and Na^+^/H^+^-PPases are only active in the presence of Na^+^ or Li^+^ (refs 1, 9), which are the two pumped alkali cations (Na^+^ being the physiologically relevant one). In the presence of Na^+^, K^+^ activates the enzyme and increases the enzyme's affinity for Na^+^ by about 200 fold (*K*_m_^Na+^ 9–80 mM in the absence of K^+^ and 0.036–0.45 mM in its presence^1^,^4^,^9^,^10^).

So far, the structures of two M-PPases have been solved in three different states: TmPPase in resting and product-bound states (TmPPase:Ca:Mg and TmPPase:P_i2_, respectively)^11^ and VrPPase in a substrate-analogue imidodiphosphate (IDP)-bound state (VrPPase:IDP)^12^. These structures have shown that M-PPases are homodimers, with each protomer having 16 transmembrane helices (TMHs) and a vertically arranged, continuous active site structure with four distinctive parts: ‘hydrolytic centre', ‘coupling funnel', ‘ion gate' and ‘exit channel'^3^ (Fig. 1).

The hydrolytic centre is situated on the cytoplasmic side of the protein with conserved aspartates and lysines lining the binding pocket and coordinating the binding of Mg^2+^, IDP and a water molecule that is poised for nucleophilic attack^12^. The closure of the active site cavity by the loop between TMHs 5 and 6 (loop 5–6) upon substrate binding is necessary for directional pumping^12^. On the periplasmic/lumenal side of the hydrolytic centre are the coupling funnel, ion gate and exit channel (Fig. 1).

Mutagenesis of key residues in TMH12 uncouples hydrolysis and pumping^13^,^14^, while mutagenesis of the gate residues *E*^6.53/6.57^ (the aforementioned semi-conserved glutamate), *S*^6.54^ and *D*/*N*^16.46^ are important for sodium binding in Na^+^-PPases and for proton-pumping activity in H^+^-PPases^8^,^12^,^14^,^15^,^16^. This suggests that the sodium-binding site of Na^+^-PPases is found near these residues^16^ with a lack of Na^+^ affecting hydrolysis, but not Mg_2_PP_i_ binding^4^.

Clearly, the starting point of ion pumping is the nucleophilic water at the hydrolytic centre^12^, which loses a proton upon electrophilic attack on the PP_i_. Three different mechanisms have been proposed for how pumping is coupled to hydrolysis: substrate binding^11^, hydrolysis^12^ or a ‘Mitchell-direct' mechanism, where the proton released during hydrolysis is directly pumped^17^. The first two mechanisms differ in their timing: does hydrolysis precede pumping or not? The third mechanism, as it says that the proton released during hydrolysis is pumped, is not easy to reconcile with Na^+^-pumping enzymes. The other major question is the role of the coupling funnel: how does it connect proton abstraction from the water nucleophile to ion pumping—and indeed pumping of a different ion?

In this article we present three structures: the Na^+^-pumping TmPPase with IDP and Na^+^ bound (TmPPase:IDP) at 3.5 Å resolution, the H^+^-pumping VrPPase with one phosphate bound (VrPPase:P_i_) at 3.5 Å resolution, and the newly refined structure of TmPPase with the phosphate analogue, WO_4_ bound, at 4 Å resolution (TmPPase:WO_4_). (This last was previously used to solve the phase problem in the original TmPPase structure, but the structure was not refined^11^.) We suggest where and how TmPPase binds Na^+^, and how H^+^-pumping evolved from Na^+^-pumping. We develop a plausible unified mechanism for how substrate binding leads to nucleophile activation and ion pumping and provide electrometric evidence that suggests pumping precedes hydrolysis upon substrate binding. Finally, we suggest that the atomic mechanism of gating in M-PPases is similar to that of the ATPases.

## Results

### Structural overview

The overall structures of TmPPase:IDP, TmPPase:WO_4_ and VrPPase:P_i_ are similar to our previous structures^11^,^12^ with root mean squared deviation (r.m.s.d.) values of 0.7 Å for TmPPase:IDP-TmPPase:P_i2_, 1.6 Å for TmPPase:IDP-TmPPase:Ca:Mg and 1.0 Å for VrPPase:P_i_-VrPPase:IDP (Supplementary Fig. 1). The former was solved by molecular replacement against protein data bank (PDB): 4AV3 (ref. 11) at 3.5 Å and the latter by molecular replacement against PDB: 4A01 (ref. 12) at 3.5 Å (Table 1, see ‘Methods' section and Table 2).

**Figure 2:**
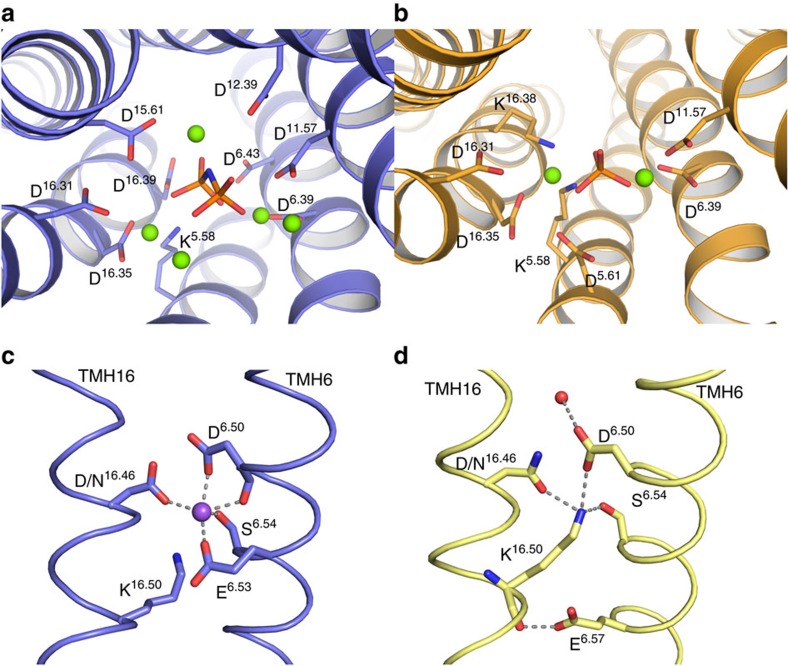
Hydrolytic centre and ion gate of TmPPase and VrPPase. Key residues are labelled and hydrogen bonds shown as dashed lines. (**a**) The hydrolytic centre of TmPPase:IDP (blue in all figures) with bound Mg^2+^ (green), IDP (orange) and coordinating residues. (**b**) Hydrolytic centre of VrPPase:P_i_ (gold) showing Mg^2+^ (green), P_i_ (orange) and coordinating residues. (**c**) Ion gate region of TmPPase:IDP showing bound Na^+^ (purple) and its coordination (grey). TMH 6 and 16 are labelled. (**d**) Ion gate of VrPPase:IDP (yellow) showing the salt-bridge network (yellow) and coordinated waters (red). TMH 6 and 16 are labelled.

In TmPPase:IDP, an Mg_5_IDP-complex was fit into the positive F_o_–F_c_ density in the hydrolytic centre (Supplementary Fig. 2a) and then refined. The coordination of Mg^2+^ and IDP is almost identical to that seen in VrPPase:IDP^12^ (Fig. 2a and Supplementary Fig. 3), even though the two enzymes pump different ions. In VrPPase:P_i_, the extra F_o_–F_c_ electron density in the PP_i_-binding pocket is a phosphate ion, just 0.6 Å from the leaving group phosphate in VrPPase:IDP (Supplementary Fig. 2d). This phosphate ion is coordinated by *K*^5.58^, *K*^16.38^ and two Mg^2+^ ions, which are situated in a similar position to Mg2 and Mg3 in VrPPase:IDP and to the Ca^2+^ and Mg^2+^ in resting-state TmPPase (Fig. 2b).

### Bound sodium ion

Positive F_o_–F_c_ density was seen in TmPPase:IDP between residues *D*^6.50^, *E*^6.53^, *S*^6.54^ and *D*^16.46^ (Fig. 2c and Supplementary Fig. 2b). Close to this, *K*^16.50^, which forms an ion-triplet in the resting-state TmPPase structure with *D*^6.50^ and *E*^6.53^, adopts a different orientation and the *K*^16.50^ Nζ is disordered. Mutagenesis has shown that the gate residues *E*^6.53^, *S*^6.54^ and *D*/*N*^16.46^ are important in sodium binding in Na^+^-PPases^16^. A sodium ion was therefore refined into this density. The resulting bond distances were between 2.39–2.49 Å (mean: 2.43 Å) and the site was penta-coordinated (by the carboxylate groups of *D*^6.50^, *E*^6.53^ and *D*^16.46^, the O^*γ*^ of *S*^6.54^ and the main-chain carbonyl of *D*^6.50^)^18^ (Fig. 2c). A score of 0.90 was obtained from a valence test^19^ at this position, with scores over 0.7 indicative of sodium ions (scores were between 0.1 and 0.4 for water molecules elsewhere in the structure). All of these results indicate that the density is most likely a bound Na^+^ and not a water molecule.

### Comparison with previous structures

**Figure 3:**
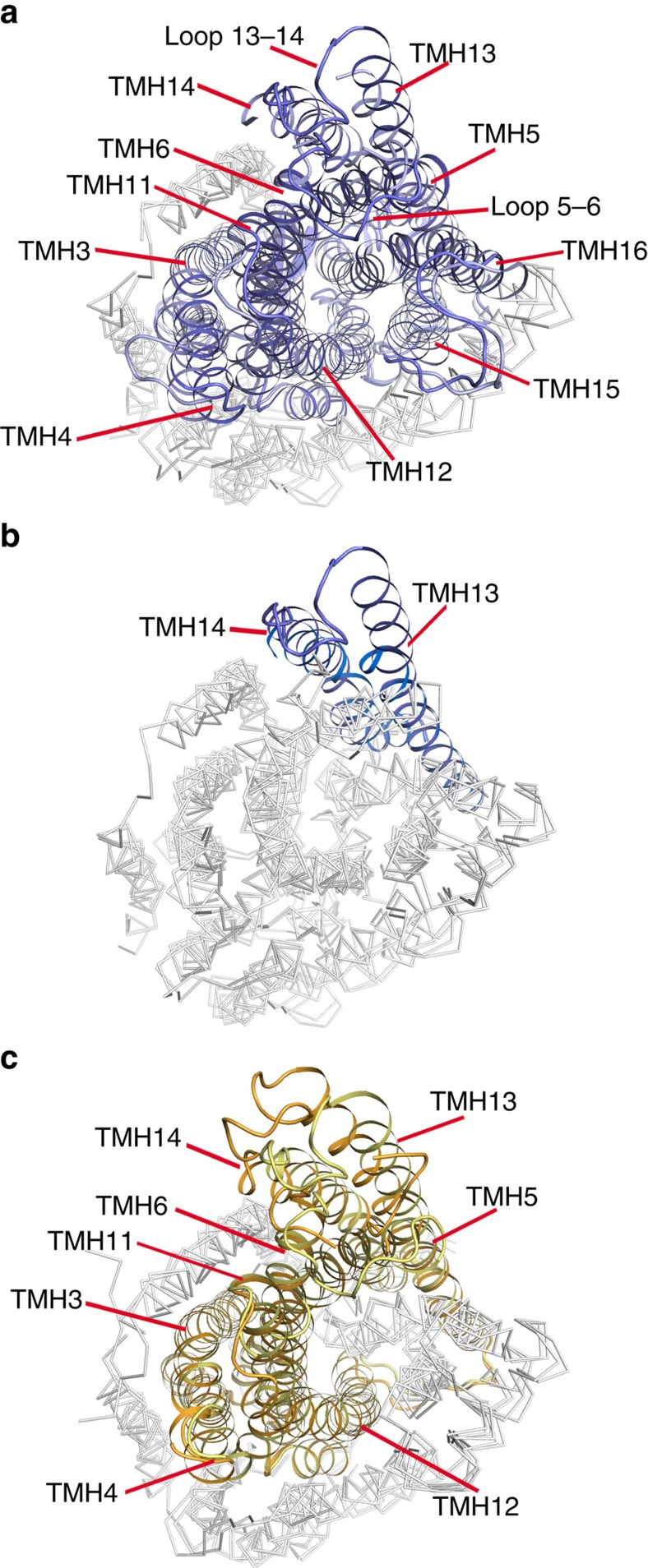
Movements associated with constriction and opening of the active site. (**a**) Comparison of TmPPase:IDP (blue) and TmPPase:Ca:Mg (light blue). TMHs 3, 4, 5, 6, 11, 12, 13, 14, 15 and 16, which move between these two states are labelled and coloured, while the rest is in grey. Loops 5–6 and 13–14, which are visible in TmPPase:IDP but not in TmPPase:Ca:Mg, are also labelled. (**b**) Comparison of TmPPase:IDP and TmPPase:P_i2_ (sky blue). Moving TMHs 13 and 14 are labelled and coloured. (**c**) Comparison of VrPPase:IDP (yellow) and VrPPase:P_i_ (gold). Moving TMHs 3, 4, 5, 6, 11, 12, 13 and 14 are coloured and labelled. The r.m.s.d./C*α* are: resting TmPPase:Ca:Mg-TmPPase:IDP, 1.70 Å; TmPPase:Ca:Mg-TmPPase:P_i2_, 1.31 Å; TmPPase:IDP-TmPPase:P_i2_, 0.90 Å; VrPPase:P_i_:VrPPase:IDP, 1.12 Å.

The new structures of TmPPase and VrPPase were compared with previously solved structures (PDB ID: 4AV3, 4AV6, 4A01). Notable was the volume of the hydrolytic centre, which approximately halves upon binding substrate: from 3,400 Å^3^ in resting-state TmPPase to 1,600 Å^3^ in the IDP-bound states of VrPPase and TmPPase, but expanding to 2,800 Å^3^ in VrPPase:P_i_ (Table 1). The cytoplasmic ends of the inner-ring TMHs 5, 6, 11, 12, 15 and 16 and the outer-ring TMHs 3, 4, 13 and 14 are constricted in TmPPase:IDP^12^ (Fig. 3) and loops 5–6 and 13–14 are ordered and cover the active site cavity. The closure of the active site cavity involves the formation of an intricate salt-bridge network between the residues of TMHs 5, 12, 15 and loop 5–6 in both VrPPase:IDP^12^ and TmPPase:IDP. Loop 13–14, which folds on top of loop 5–6, probably plays an important role in stabilizing the placement of loop 5–6, and hence the closure of the active site, as only TMHs 13 and 14 move horizontally during the conversion from the IDP- to P_i2_-bound states (Fig. 3b).

The largest movement in the entire structure upon substrate binding is at TMH13 (5.5–8 Å at the C*α*), apparently driven by motion at TMH5 and loop 5–6 (Supplementary Fig. 4a). The interactions of *E*^5.71^–*R*^13.62^–*R*/*I*^10.33^, are abolished in the absence of substrate (Supplementary Fig. 4b,c); *R*^13.62^ links motions of the inner ring to the outer ring and into the neighbouring protomer. This motion is not propagated through the membrane: the membrane and periplasmic regions of TMHs 10 and 13 do not show any sign of conformational change. Nonetheless, it is this region that contributes most to the M-PPase dimer interface^12^.

### Analysis of helical and hydrogen bond geometry

The region surrounding *D*^6.43^ in TmPPase:IDP has approximately canonical *α*-helical hydrogen-bonding (calculated using HBplot (VirtuaDrug, Hungary)^20^,^21^), whereas the structures are significantly bent in the resting state (Supplementary Table 2). Residues around *D*^6.43^ bend on average 15° from linear as measured using Bendix^22^ (Supplementary Fig. 5). When TMH12 moves down by 2 Å, the loss of the K^12.50^–D^6.43^ ion pair leads to straightening of the 6.43 region by about 7° and a spring-loaded 3–4 Å movement of the entire *D*^6.43^ carboxylate group so that it now hydrogen bonds the water nucleophile, as seen in the VrPPase:IDP structure (Supplementary Fig. 6d). Because of this conformational change, some of the hydrogen bonds are lost and others converted to a 3_10_ pattern before *D*^6.43^, with a *π*-helix hydrogen-bonding pattern after it (Supplementary Table 2). In addition, the backbone carbonyl on *D*^6.43^ is flipped away from the helix axis and can no longer make a hydrogen bond to the backbone amide on *L*/*M*^6.47^ (Supplementary Table 2).

In addition to the changes seen in TMH 6 (above), TMH 16 also changes conformation when IDP binds (Supplementary Figs 5 and 7), with ∼1.5 Å ‘corkscrew change' in TMH16 at N/D^16.46^–K^16.50^ due to the formation of a few residues of 3_10_ helix at *S*^16.44^–*L*^16.45^ (Supplementary Table 2). These helical movements shift the positions of *D*^6.50^ and *S*^6.54^ in VrPPase (Fig. 2d and Supplementary Fig. 7b) and of *D*^6.50^, *E*^6.53^, *S*^6.54^ and K^16.50^ in TmPPase (Fig. 2c and Supplementary Fig. 7a).

### Electrometric studies of proton pumping

We studied the pumping of H^+^ by VrPPase on the Nanion SURFE^2^R N1. This technique utilizes a membrane-coated, gold sensor on individual chips. The protein is first reconstituted into liposomes (see ‘Methods' section), the liposome is then adhered to this membrane layer and the current across the liposomes is measured over time following a rapid switch between two ionically balanced buffers, with the activating buffer containing the relevant substrate or inhibitor.

**Figure 4:**
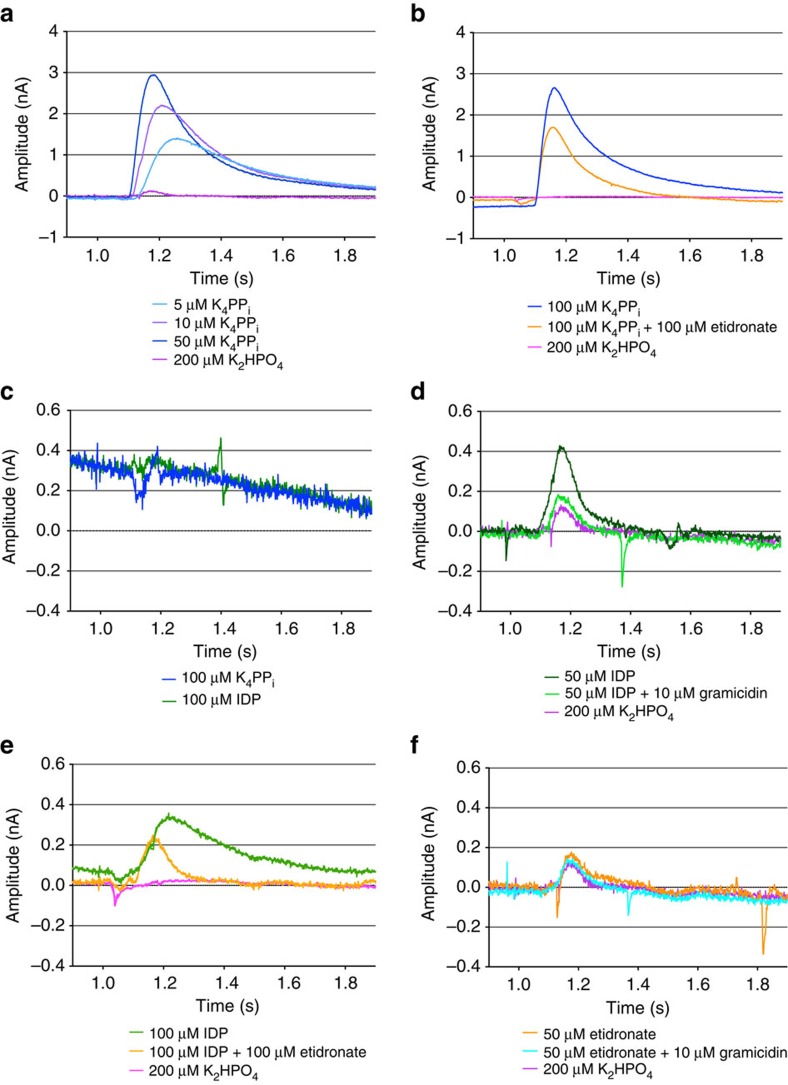
Electrometric sensor traces for H^+^-pumping. Traces obtained for VrPPase from the Nanion SURFE^2^R N1 machine. (**a**) Effect of increasing concentrations of K_4_PP_i_ and K_2_HPO_4_ on signal amplitude of VrPPase proteoliposomes. (**b**) Effect of etidronate addition to K_4_PP_i_-induced signals of VrPPase proteoliposomes. (**c**) Effect of K_4_PP_i_ and IDP on liposomes without pyrophosphatase. (**d**) Effect of IDP on amplitude in the presence and absence of gramicidin compared with K_2_HPO_4_. (**e**) Effect of etidronate addition to IDP-induced signals of VrPPase proteoliposomes. (**f**) Effect of etidronate on amplitude in the presence and absence of gramicidin compared with K_2_HPO_4_. Activating buffer containing substrate or inhibitor was added at 1 s and removed at 2 s. Two anomalous peaks (covering <0.01 s) were removed from **e** for the sake of clarity.

A positive signal up to 3 nA is repeatedly observed with K_4_PP_i_ (Fig. 4a). This signal is diminished when the inhibitor etidronate (Ethane-1-hydroxy-1,1-diphosphonate; PO_3_–C(CH_3_)(OH)–PO_3_) is added alongside K_4_PP_i_ (Fig. 4b) and absent in control sensors without protein (Fig. 4c) or with K_2_HPO_4_ (Fig. 4a; around 0.2 nA). A signal of 0.4 nA is observed in the presence of only IDP, twice as strong as that from K_2_HPO_4_ (Fig. 4d). This signal was significantly reduced in the presence of 10 μM gramicidin (Fig. 4d) and etidronate (Fig. 4e), in both cases to about 0.1–0.2 nA, similar to the signal from adding K_2_HPO_4_.

## Discussion

Our new IDP- and single-phosphate-bound structures provide a clear model of how substrate binding leads to nucleophile activation (Supplementary Fig. 6b,d), as we can now compare multiple structures from the same protein (Table 1) and at higher resolutions. As the protomers in each structure are the same (r.m.s.d.=0.1–0.4 Å), we discuss just protomer A below.

Mutations in six interacting residues (*V*^10.40^A, *I*^10.44^T, *L*^10.48^P, *V*^10.52^I, *F*^13.40^*L*, *L*^13.41^Q) in the dimer interface of *Streptomyces coelicolor* PPase either led to an inactive enzyme or one where PP_i_ hydrolysis and pumping were not tightly coupled^15^ (Supplementary Table 3). These residues are neither part of the hydrolytic centre, the coupling funnel nor of the ion gate^3^,^11^,^12^. They nonetheless interact in all states currently observed, especially *M*/*Y*^10.48^ and *Y*^13.40^ (Supplementary Fig. 4b,c).

Furthermore, interactions across the protomer–protomer interface might explain the linking of Na^+^ and H^+^ pumping in the dual-pumping Na^+^/H^+^-PPases^1^. This is otherwise difficult to explain, as there are no obvious structural differences in the channel compared with other M-PPases and no secondary channels have been identified. We therefore speculate that there is an allosterically driven dual catalytic cycle, where pumping of a proton by protomer ‘A' causes a conformational change in protomer ‘B' that enables Na^+^-pumping and vice versa, although other possibilities cannot be excluded.

All hydrolytic enzymes can only be activated upon binding of the correct substrate to prevent hydrolysis of related molecules^23^. M-PPases, for instance, could potentially hydrolyze the entire ATP pool. Our new model defines the structural basis for substrate specificity and explains how the water nucleophile is activated when the cognate substrate binds. We earlier noticed that TMH12 moved ‘down' by about 2 Å upon substrate binding^11^ and appears to be linked to breakage of the salt-bridge between K^12.50^ and *D*^16.39^, which coordinates the nucleophilic water^3^. However, the observation did not explain hydrolysis site activation, and could have been an artefact because we compared two different proteins and one structure was at just 4 Å resolution.

A key feature of all the open states is that *D*^6.43^ is pointed away from the nucleophilic water because it forms an ion pair with K^12.50^ (Supplementary Fig. 6b,d). As a result, the nucleophilic water is activated by *D*^16.39^ alone, and so the enzyme is inactive. Upon binding of the correct substrate, closure of the active site by TMHs 5 and 13 leads to a more than 2 Å downward motion at TMH12, seen in all open→closed comparisons (for example, TmPPase:Ca:Mg→TmPPase:IDP; Supplementary Fig. 6a,c). This leads to activation of the nucleophilic water by both *D*^6.43^ and *D*^16.39^ due to the rotation and reconfiguration of TMH 6.

Using the bendix (Supplementary Fig. 5) and HBplot data (Supplementary Table 2) we propose that this corresponds to the springing of a ‘mousetrap', triggered by the arrival of the ‘mouse'—the pyrophosphate. Motion at TMH12 breaks the K^12.50^–D^6.43^ ion pair, forming the activated *D*^6.43^–H_2_O–D^16.39^ nucleophile. Significant free energy seems to be available, as the resultant helical conformation becomes less ideal. This can only happen upon binding of correct substrate: polyphosphate and nucleoside di- and tri-phosphates are too large and prevent closure of the active site as, indeed, appears to be the case with etidronate as well (see below). We suggest that springing of the ‘molecular mousetrap' is fast so that the requisite sequencing of events on the enzyme will occur, and hydrolysis and pumping are coupled; binding will drive pumping and thus hydrolysis. However, a key question remains: how does this model explain ion selectivity?

In the open states, K^16.50^ forms an ion-pair with the semi-conserved glutamate (E^6.53^ in TmPPase and *E*^6.57^ in VrPPase) at the ion gate. In the IDP-bound states of both proteins, two notable changes occur: the salt bridge is broken and *D*/N^16.46^ swings into the vicinity of the other gate residues (Supplementary Fig. 7). The big difference between TmPPase and VrPPase is in the position of K^16.50^; indeed this is the most significant difference in the entire active site and so must be related to the central difference between the proteins—their pumping specificity. In TmPPase:IDP (Fig. 2c), it has swung away and is disordered (Nζ B-factor=112.5 Å^2^), while in VrPPase:IDP, K^16.50^ forms a well-ordered ion pair with *D*^6.50^ (Fig. 2d). This difference is intimately related both to coupling and to ion selectivity, as we describe below.

In TmPPase, sodium binding is facilitated by the inward motion of *D*^16.46^ into the sodium-binding site and by the movement of TMHs 6 and 16, which is almost twice as big as that in VrPPase. These helical movements bring *D*^6.50^, *E*^6.53^ and *S*^6.54^ to the correct position to bind Na^+^ and move K^16.50^ away from the binding site (Supplementary Fig. 7a). The changes are, of course, driven by the downward motion of TMH 12 and the loss of the *D*^6.43^–K^12.50^ ion pair, and so by substrate binding and water activation (see above; Supplementary Fig. 7a). This also provides a direct link between substrate and ion binding: the Na^+^ site forms only upon substrate binding, and so the binding affinity of substrate increases in the presence of sodium and vice versa^4^.

By contrast, VrPPase has a completely different gate structure due to the shift in the position of the semi-conserved glutamate. The VrPPase:IDP has, instead of Na^+^, a number of water molecules lining the channel, including one coordinated by the gate residues (Fig. 2d). These water molecules are absent in the VrPPase:P_i_, probably due to the low resolution of the structure (3.5 Å). Nonetheless, K^16.50^ is ordered in both states of VrPPase, unlike in TmPPase. We propose that, in the IDP-bound state, the semi-conserved glutamate *E*^6.57^ at the ion gate is protonated, disrupting the *E*^6.57^–K^16.50^ ion pair, and causing K^16.50^ to move ‘upward' (Supplementary Fig. 7). This leads to charge separation at the gate, with a full-proton localized on *E*^6.57^, the ionic gate closed and K^16.50^ neutralized by *D*^6.50^, *S*^6.54^ and N^16.46^ (Fig. 2d) in a way that is analogous to the neutralization of the charge of Na^+^ in TmPPase (Fig. 2c).

The description above has another fundamental implication: in TmPPase, the positive charge to be pumped (the sodium ion) is still above K^16.50^ (on the cytoplasmic side; Fig. 2c), whereas in VrPPase, it is below (the proton localized on *E*^6.57^) and thus ready to be transferred to the exit channel. It is nonetheless likely that both states (charge above K^16.50^ and charge below K^16.50^) exist in both proteins. Our structures thus appear to visualize two different sub-states in the catalytic cycle.

Finally, functional experiments with *Streptomyces coelicolor* M-PPase have shown that M256^6.47^T and I242^6.33^T mutant proteins have 90–100% of wild-type hydrolysis activity, but only a 25–30% wild-type coupling ratio between hydrolysis and proton-pumping^15^. Even though the residues are not completely conserved (VrPPase: *M*^6.47^ and *I*^6.33^; TmPPase: *L*^6.47^ and *I*^6.33^) the fact that these are hydrophobic to polar mutations create ‘loose' coupling mutations is consistent with our model that TMH6 is also essential in ensuring coupling. These mutations could perturb the movement of this helix so that hydrolysis can proceed even without ion transport to the exit channel.

**Figure 5:**
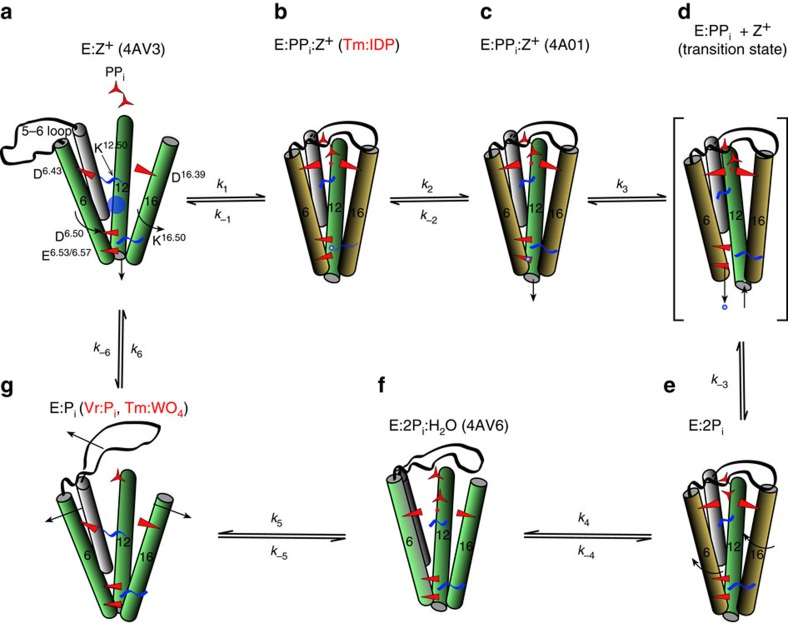
Complete kinetic scheme of M-PPase catalytic cycle. (**a**–**g**) All the proposed conformational states of the M-PPase catalytic cycle are shown in order, including the transition state for pumping (**d**) and a ‘relaxed product' state (**f**) analogous to yeast pyrophosphatase^46^. The structures used in the manuscript are shown, with the new structures marked in red; for the rest, the PDB code is shown. For clarity, only key helices involved in formation of the hydrolytic centre and ion channel are displayed. Aspartate and lysine residues are shown in red and blue respectively and labelled on state (**a**). Changes to helix position and rotation are denoted with arrows and by changes in the shading of the helices. The pumped ion (Na^+^/H^+^) is represented as a blue sphere in states (**b**–**d**) and as a large blue sphere in state (**a**) to denote the unknown position of the pumped ion in the channel of the resting state. The nucleophilic water is represented as a red sphere in states (**b**–**d**).

The structures described in Table 1 encompass all the states for a minimal catalytic scheme for M-PPases (Fig. 5). For a pump to achieve directional flow, the sequence of events—that is, the rates of the unitary steps—must be organized such that pumping is faster than the reverse reaction. For M-PPases, it implies that, for example, *k*_3_ must be faster than *k*_−2_ for unitary committing steps on the enzyme and, typically, that *k*_3_<*k*_2_<*k*_1_ (Fig. 5) as has been seen, for instance, in cytochrome c oxidase^24^,^25^. This ensures that H^+^/Na^+^ pumping is faster than opening to the cytoplasm and release of PP_i_ or P_i_. Our static structures can only hint at the possibilities, but we believe that the key step is the breaking of the *D*^6.43^–K^12.50^ ion pair. This appears to release stored strain energy in the structure, as it leads to a new conformation where the *D*^6.43^ carboxylate group moves towards the now-bound negatively charged phosphate (Fig. 5b), the 6.43 region does not form main-chain hydrogen bonds, and the pumped ion becomes localized at the ionic gate. Consistent with this, Lee *et al*.^26^ have shown that mutation of K^12.50^ in VrPPase abolishes both hydrolysis and pumping. The role we propose explains these results as K^12.50^ does not bind substrate, nor is it part of the ionic gate.

We therefore speculate that the pyrophosphate binding and localization of the ion is the fast step in the reaction cycle and that the reverse of this step is slow (*k*_1_>*k*_−1_). In addition, we propose that TmPPase:IDP and VrPPase:IDP structures represent two different states in the pumping process: the former with the pumped ion cytoplasmic (above K^16.50^; Fig. 5b), and the latter with it lumenal (below K^16.50^; Fig. 5c). At this point, the protein is open neither to the cytoplasm nor to the periplasmic/lumenal side; and the gate is closed. The next, presumably slower step, would involve the exit channel opening, probably through further ‘downward' movement of TMH12 (ref. 11), diffusion of the ion away and closure of the gate (Fig. 5d). This would pave the way for hydrolysis as the final, slow-committing step (Fig. 5e). Only after these steps have happened can the active site reopen on the cytoplasmic side, and the two phosphates leave in an ordered manner (Fig. 5f,g; see above). This proposed mechanism thus ensures coupling between hydrolysis and pumping. The opening of the active site presumably also decreases the affinity of the active site for product (MgP_i_), allowing the second phosphate to leave the active site, and thus returning the enzyme to the resting state (M-PPase:Mg_2_; Fig. 5a). A similar kinetic scheme run in reverse would explain how M-PPases are able to synthesize pyrophosphate. This raises another important question: which comes first, hydrolysis or pumping?

The best possible explanation of the 0.4 nA signal from IDP (Fig. 4d) from the Nanion SURFE^2^R results is that this non-hydrolyzable analogue causes a single-turnover event as substrate hydrolysis is essential for continued ion pumping; without it, product release cannot occur. This would prove that substrate binding drives pumping, the model we prefer, as pumping can occur without hydrolysis.

This is supported by three lines of argument. First, gramicidin forms a specific channel for monovalent cations. Any reduction in the proton-driven signal from IDP upon adding gramicidin must therefore be due to a collapse of the monovalent cation gradient. As there is a clear and reproducible reduction upon adding gramicidin (Fig. 4d), IDP must cause proton pumping. Second, we have a clear measure of the component of the current due to binding of Mg^2+^ to the protein. The residual signal (0.1–0.2 nA), seen when etidronate or KH_2_PO_4_ (Fig. 4) bind, can not be collapsed by gramicidin (Fig. 4f), and so is due to this other component. The Nanion SURFE^2^R data thus also suggest that IDP and etidronate bind differently; IDP binds like substrate and is able to drive the conformational changes required for ion-pumping, while etidronate is not able to bind in the same manner. This is so even though etidronate is a somewhat better inhibitor of VrPPase^27^ (IDP *K*_i_=12 μM, Etidronate *K*_i_=6.5 μM). Modelling etidronate into the active site indicates the following: it prevents proper closure of the active site due to steric hindrance with *D*^15.61^ and one of the coordinating magnesium ions (Supplementary Fig. 8). Third, Fig. 4a,d provides further evidence that there is coupling between substrate hydrolysis and ion pumping in the catalytic cycle of M-PPases, since the signal induced by PP_i_ (1–3 nA) is up to ten times greater than that induced by IDP (0.4 nA). Conversely, we suggest that etidronate prevents the formation of vital interactions in the hydrolytic centre or closure of the active site, so neither pumping nor hydrolysis can occur. This again emphasizes the importance of substrate specificity to enzyme activation.

It cannot be fully excluded that the single-turnover signals are due to alternative transport events. We again note that the gramicidin control (Fig. 4d) strongly suggests that IDP, contrary to etidronate, induces a transmembrane charge transfer. If this charge transfer is not connected to the pumped proton as we propose here, alternative transport mechanisms could be envisioned in which the proton is transported after PPi. This would be similar to the proposal in Lin *et al*.^12^, but not similar to the established mechanism in rotary ATPases^28^,^29^, nor to the general behaviour of non-control point enzymes, where the free energy difference between substrate and product is expressed as the free energy difference between the binding of substrate versus the binding of product^30^.

Recent mutagenesis studies^14^ showed that the *I*^12.54^A and *L*^12.64^A (Supplementary Fig. 6c) mutants of VrPPase are decoupled: they do not pump but retain half of the hydrolysis activity of wild-type. This corroborates our hypothesis, as these mutants would change the molecular smoothness of the lower-end of TMH12, thus affecting its downward motion^11^.

**Figure 6:**
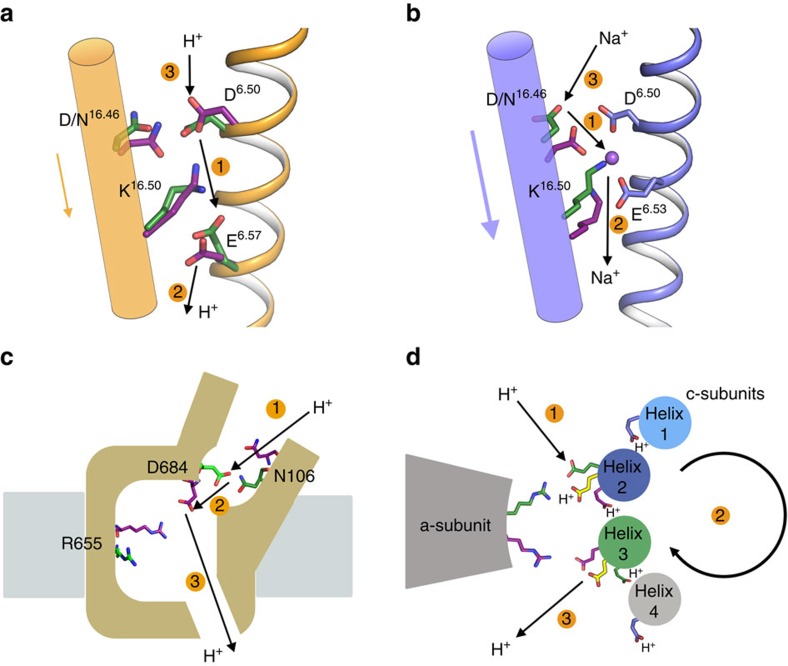
Comparison of M-PPases with ATPases, demonstrating the formal similarity and the coupling of Lys/Arg motion to ion pumping. (**a**) Proton pumping and peristaltic motion of K^16.50^ in VrPPase and (**b**) sodium pumping and peristaltic motion of K^16.50^ in TmPPase. (**c**) Structural overview and hypothesized proton transport mechanism of P-type ATPases^31^. (**d**) Proton pumping and rotary motion in F-ATPase^29^: (based on figure 6 from reference 29) in the hydrolysis direction. Colour scheme: green, initial residue states; yellow, intermediate states; magenta, final states; blue before and after (F-ATPase). *D*^6.50^ and *E*^6.53^ in (**b**) and the helices in (**a**,**b**) are coloured as in Figs 2 and 3 to emphasize the fact that the motions there are relatively small. Numbers represent order of ion progression through the respective ion channels.

There is neither sequence, structure nor overall mechanistic similarity between M-PPases and the pumping ATPases^11^,^12^. We believe, however, that there is nonetheless distant mechanistic similarity at the ion gate. The protonated-gate state of VrPPase:IDP (Fig. 6a) and the ion-bound state of TmPPase:IDP (Fig. 6b) are analogous to the ATP-analogue binding E1-state of P-type H^+^-ATPase^31^, where the protonated acceptor/donor residue D684 forms hydrogen bonds with N106 inside an occluded binding pocket (Fig. 6c). Similarly, in VrPPase:IDP, protonated *E*^6.57^ makes hydrogen bonds with the carbonyl group of K^16.50^ and O^*γ*^ of *S*^5.43^ but not to K^16.50^ Nζ (Figs 2d and 6a)^31^. In addition, the movement of K^16.50^ in and out of the vicinity of the proton donor/acceptor in M-PPases is like that of R210 in the F-ATPase a-subunit^29^ and R655 in P-type H^+^-ATPase^31^, where its movement is associated with ion binding and release (Fig. 6). This alternating motion may be a unifying mechanistic principle for primary ion pumps.

Phylogenetic analyses show that Na^+^-PPases were the first M-PPases to evolve and that the evolution of H^+^-PPases has occurred at least three times, leading to different H^+^-PPases with the semi-conserved glutamate in different positions (*E*^5.43^, *E*^6.53^ or *E*^6.57^)^8^. The only major difference between the gate structure of sodium-pumping TmPPase and proton-pumping VrPPase is the shift in the position of the semi-conserved glutamate from *E*^6.53^ to *E*^6.57^, respectively. While changing the position of the semi-conserved glutamate through mutagenesis has so far led only to protein inactivation^8^, our structures indicate why the *E*^6.53^ to *E*^6.57^ change led to the evolution of plant-type H^+^-PPases from Na^+^-PPases^11^.

We propose that moving the semi-conserved glutamate down one helical turn to *E*^6.57^ changes the pumping specificity first, by destroying the Na^+^-binding site in the IDP-bound state and, second, by creating a proton acceptor at the end of the cytoplasmic Grotthuss chain^32^. In the new position, it interacts with K^16.50^ in the same way as the D684–R655 pair in H^+^-ATPase (see above). As a result, K^16.50^ Nζ adopts the same conformation in the IDP-bound H^+^-PPases (Supplementary Fig. 7b) as it does in the resting state of Na^+^-PPases (Supplementary Fig. 7a), leading to the change in pumping specificity. The gate residues, which in Na^+^-PPases would bind Na^+^, neutralize K^16.50^: slight changes in the order of conformational steps lead to a profound change in the ion pumped.

The model also explains the evolution of Na^+^/H^+^-pumping M-PPases (see above). If this model is correct, the switch between pumping Na^+^ and H^+^ only requires repositioning TMH6 so that the Na^+^ site does not form, but that *E*^6.53^ can act as the end of the Grotthuss chain. This could be driven by the allosteric motion driven by TMH13, as described above. We are exploring these ideas.

## Methods

### Expression and purification of VrPPase

*Saccharomyces cerevisiae* strain BJ2168 was transformed with the galactose-inducible vector pYES2 containing C-terminally His_6_-tagged VrPPase by the lithium acetate transformation method^33^. All yeast cells were collected by centrifuging at × 2,300*g* at 4 ° C for 10 min after 3-day induction. Next, the yeast cells were resuspended in 5 mM Tris/Ethylene glycol tetraacetic acid (EGTA) (pH 7.6), 10% (w/v) glycerol, 0.6% (w/v) Tris (base), 0.6% (w/v) ascorbate, 1.5% (w/v) Polyvinylpyrollidone (*M*_r_ 40,000; PVP40000), 1 mM Phenylmethylsulfonyl fluoride (PMSF) and 1 μg ml^−1^ pepstatin A, and disrupted by ultra-sonication. The homogenate was then centrifuged differentially at 2,300*g* for 10 min and 126,000*g* for 35 min at 4 ° C. After ultracentrifugation, the membrane vesicles were resuspended in extraction buffer (25 mM MOPS/KOH (pH 7.0), 400 mM KCl, 4 mM MgCl_2_, 20% (w/v) glycerol and 1 mM PMSF) and solubilized by using *n*-dodecyl-β-D-maltopyranoside at a protein-to-detergent ratio of 1:2.5. The suspension was gently stirred at 4 ° C for 1 h followed by centrifugation at 126,000*g* for 35 min. After ultracentrifugation, the solubilized VrPPase was loaded onto a Ni^2+^-NTA column and eluted with a buffer containing 25 mM MOPS/KOH (pH 7.1), 400 mM KCl, 4 mM MgCl_2_, 20% (w/v) glycerol, 1 mM PMSF, 0.15% (w/v) *n*-decyl-β-D-maltopyranoside and 250 mM imidazole. The purified VrPPase was exchanged to the crystallization buffer (25 mM 4-morpholineethanesulfonic acid (MES) (pH 6.5), 400 mM KCl, 4 mM MgCl_2_, 20% (w/v) glycerol, 0.15% (w/v) *n*-decyl-β-D-maltopyranoside) and concentrated to 10 mg ml^−1^ for crystallization.

### Expression and purification of TmPPase

The expression and purification of TmPPase followed a modified version of the ‘hot-solve'-purification method^34^. BJ1991-strain (Mat(Δ) leu2 trp1 ura3–251 prb1–1122 pep4-3 gal2) *Saccharomyces cerevisiae* cells were transformed with plasmid pRS1024, which contained a LEU2-gene and N-terminally His_6_-tagged TmPPase under a constitutive PMA1-promoter^34^. The cells were first grown in selective synthetic complete drop-out (SCD)-media to the end of the exponential growth phase and then 250 ml of this culture was used to inoculate 740 ml of 1.5 × concentrated YEP-media with 2% glucose. The cells were grown in this for 8 h at 30 °C after which they were collected, lysed via sonication with 0.2 mm glass beads and their membranes were extracted using ultracentrifugation (100,000*g* for 45 min) with pellets resuspended in resuspension buffer: 50 mM MES-NaOH pH 6.5, 20% (v/v) glycerol, 50 mM KCl, 5.2 mM MgCl_2_, 0.33 mM Na_2_PP_i_, 1.33 mM Dithiothreitol (DTT), 2 μg ml^−1^ (w/v) pepstatin-A (Sigma) and 0.334 mM PMSF (Sigma)^34^. For solubilization, the extracted membranes were diluted into 30 ml fractions containing 7.2 mg ml^−1^ of membranes in resuspension buffer and heated at 75 °C for 15 min. After this each membrane aliquot was mixed with 10 ml of pre-heated solubilization buffer containing 50 mM MES-NaOH pH 6.5, 20% (v/v) glycerol and 5.334% (w/v) *n*-dodecyl-β-D-maltopyranoside (Anatrace) and incubated at 75 °C for 1.5 h. The denatured proteins were removed by immediately centrifuging at 3,300*g* for 5 min at 22 °C and then at 3,300*g* for 15 min at 4 °C. KCl was added to a final concentration of 0.3 M together with 2 ml of Ni-NTA matrix (Qiagen) to each 40 ml fraction of solubilized protein. The protein was bound to the Ni-NTA matrix at 42 ° C for 1.5 h after which the matrix was loaded into a column. The column was washed with 1.5 × column volume (CV) of washing buffer (50 mM MES-NaOH pH 6.5, 20% (v/v) glycerol, 50 mM KCl, 20 mM imidazole pH 6.5, 5 mM MgCl_2_, 1 mM DTT, 2 μg ml^−1^ (w/v) pepstatin-A, 0.2 mM PMSF and 0.5% octyl glucose neopentyl glycol (OGNPG, Anatrace)) and the protein was eluted with 2 × CV of elution buffer (50 mM MES-NaOH pH 6.5, 3.5% (v/v) glycerol, 50 mM KCl, 400 mM imidazole pH 6.5, 5 mM MgCl_2_, 1 mM DTT, 2 μg ml^−1^ (w/v) pepstatin-A, 0.2 mM PMSF and 0.5% OGNPG).

### Crystallization and structure determination of VrPPase

Crystallization trials were carried out using the hanging-drop vapour-diffusion method^35^. The VrPPase:P_i_ complex crystals were obtained in 2 days over a reservoir solution containing 100 mM sodium/potassium phosphate (pH 6.2), 200 mM NaCl and 37% (w/v) PEG600 at 20 ° C using 0.5 μl protein drops mixed 1:1 with the reservoir solution. The X-ray diffraction data were collected at the BL44XU beamline at Spring8, Japan and BL15A1 beamline at the NSRRC, Hsinchu, Taiwan. Data were processed by HKL2000 (ref. 36). The VrPPase:P_i_ complex crystals belonged to the monoclinic space group C2 with the cell parameters *a*=225.7 Å, *b*=81.6 Å, *c*=264.8 Å and *β*=92.9° (Table 2). The Matthew's coefficient was calculated to be 3.8 Å^3^ Da^−1^, corresponding to a solvent content of 67.7% with four subunits per asymmetric unit^37^. The crystal structure was determined by molecular replacement with MOLREP^38^ using the VrPPase:IDP complex (PDB: 4A01)^12^ as a search model. There were four molecules as two dimers (AB and CD dimers) per asymmetric unit. The entire crystal structure was completed manually with COOT^39^ and refined by REFMAC5 (ref. 40). The stereochemistry of protein residues and the secondary structural features were evaluated by PROCHECK^41^. The VrPPase:P_i_ complex with two dimers per asymmetric unit contains 21,413 protein atoms, four P_i_ and eight Mg^2+^ ions. The structure was refined to an *R*-factor of 22.6% and an *R*_free_ of 30.4% at 3.5 Å. The data collection and refinement statistics are listed in Table 2 and representative electron density for the P_i_ in Supplementary Fig. 2d.

### Crystallization and structure determination of TmPPase

For use in crystallization, the purified TmPPase was concentrated with Amicon Ultra 50,000 MWCO concentrator (Millipore), buffer exchanged to crystallization buffer (50 mM MES-NaOH pH 6.5, 3.5% (v/v) glycerol, 50 mM KCl, 5 mM MgCl_2_, 4 mM Na_4_IDP (Sigma-Aldrich: I0631), 2 mM DTT and 0.5% OGNPG) with Micro Bio-Spin 6 column (Bio-Rad) and diluted to a concentration of 10 mg ml^−1^. The crystallization of TmPPase:IDP was carried out using the vapour-diffusion method^34^ in a 1 μl+1 μl drop on a 24-well plate at room temperature using a well solution containing 32% PEG 400, 0.1 M Tris pH 8.5, 0.1 M MgCl_2_, 0.1 M NaCl and 2 mM DTT. The crystal was frozen directly from the mother liquor and X-ray diffraction data (Table 2) were collected at beamline i04 of Diamond Light Source, UK. Data for the TmPPase:WO_4_ structure were collected as part of a previous study^11^. TmPPase crystals produced as above were soaked in 10 mM Na_2_WO_4_ overnight, where they had only been used for phasing the resting TmPPase:CaMg structure in the previous study^11^. Both data sets were processed with X-ray Detector Software (XDS)^42^ and the structures were solved by molecular replacement with Phaser^43^ using the resting-state TmPPase (4AV3)^11^ as the search model. Model refinement was carried out in Phenix.refine (v. 1.9–1692)^44^, with manual alterations being made to the model in COOT^39^. Refinement was carried out at 3.5 Å and 4 Å for TmPPase:IDP and TmPPase:WO_4_, respectively, using reference restraints from the high resolution (2.6 Å) TmPPase structure (4AV3), as well as secondary structure restraints. These were used alongside Torsion-angle NCS restraints and tighter stereochemical weighting to maintain realistic geometry and to prevent over-fitting the data at these resolutions. In both models, the density for the helices was clear and unambiguous. There was additional density for the loop regions in the TmPPase:IDP structure, including the TMH 5–6 loop region. These loop regions were omitted from the TmPPase:WO_4_ structure due to the high degree of disorder in these regions. In both instances, many side chains were also omitted from the model due to a lack of density. During refinement, we saw strong positive (F_o_–F_c_) density at 4.7*σ* around the ion gate in protomer A. This was attributed to a sodium ion based upon the level of density, the optimal interaction distances with the surrounding residues and previous studies showing that mutating these residues affected sodium binding^8^,^9^,^16^. Following refinement, the *R*-factors for TmPPase:IDP were: 24.8%/27.5% with 95.4% of residues in the preferred region of the Ramachandran plot. For the TmPPase:WO_4_ structure, the *R*-factors were: 23.2%/27.9% with 93.4% of residues within the preferred region of the Ramachandran plot. Data collection and refinement statistics are listed in Table 2 with representative electron density in Supplementary Fig. 2.

### Structural analysis of TmPPase and VrPPase

We analysed all of the structures, TmPPase:IDP, TmPPase:WO_4_ and VrPPase:P_i_ (new) with the previously solved structures, TmPPase:Ca:Mg, TmPPase:2P_i_ and VrPPase:IDP (previously solved) with two pieces of software. Hydrogen bonding patterns of residues were determined and compared using the hydrogen bond plot tool HBplot (VirtuaDrug, Hungary)^20^,^21^. This information was used to determine the classification of helix (*α*, *π* or 3_10_) and was used to compare the structures. Second, the helical geometry was studied using the Bendix plugin for the Visual Molecular Dynamics software^22^. Using this software, the bend angle of each helix was calculated and compared between the structures of M-PPases.

### Reconstitution of VrPPase into liposomes

The reconstitution protocol and subsequent activity assay used were adapted from the study by Liu *et al*.^45^. Purified VrPPase protein (see above) was diluted to 50 μg ml^−1^ in VrPPase reconstitution buffer (0.25 M sorbitol, 1 mM MgSO_4_, 0.1 mM EGTA, 2 mM DTT, 10 mM Tricine-Na (pH 7.5)). 1 ml of diluted protein was mixed with 15 μl of 120 mg ml^−1^ phosphatidylcholine from soybean (dissolved in VrPPase reconstitution buffer). SM-2 Bio-beads were added at 0.25 mg μl^−1^ and the samples were incubated for 1 h at 4 °C with gentle agitation. The samples were run through Biorad micro Bio-spin 6 columns, equilibrated with VrPPase reconstitution buffer to remove residual NaCl and glycerol. Resulting proteoliposomes were frozen in liquid nitrogen and stored at −80 °C until needed for measurements. To ensure the protein was still active, the PP_i_ hydrolytic activity of the reconstituted protein was measured using the ammonium molybdate method for phosphate release^45^. The resulting activities are shown in Supplementary Fig. 9.

### Nanion SURFE^2^R measurements of VrPPase

Measurements were carried out using the SURFE^2^R N1 machine from Nanion Technologies (Munich, Germany). Sensors were prepared according to the ‘SURFE^2^R sensor preparation' protocol (Nanion's standard procedures) using their sensor prep A2 and B solutions. 0.4 μg of sonicated VrPPase proteoliposomes (UP50H compact lab homogenizer (Hielscher), 1 mm diameter tip: 20 % amplitude, 10 pulses, pulse time=0.5 s) were combined with VrPPase SURFE^2^R buffer (100 mM HEPES-KOH (pH 8), 50 mM KCl, 5 mM MgCl_2_, 0.16 mM EGTA) to a total volume of 50 μl and applied directly to each sensor. Sensors were spun at 2,500g for 30 min and stored at 4 °C for at least 1 h before measurements. Sensors were rinsed between measurements with 2 × VrPPase SURFE^2^R buffer C (100 mM HEPES-KOH, pH 8). Measurements were taken over 3 s (procedure BAB) with each buffer (VrPPase SURFE^2^R buffers A (activating buffer) and B (non-activating buffer)) added at 1 s intervals, the resulting charge differences were measured. Various compounds were added to each buffer based upon the experiments being performed (K_4_PP_i_, Na_4_PP_i_, K_2_HPO_4_, Na_2_HPO_4_, IDP, etidronate). Experiments were attempted using reconstituted TmPPase, but these were unsuccessful as TmPPase is not active enough at room temperature.

### Data availability

The atomic coordinates and structure factors of the TmPPase:IDP, TmPPase:WO_4_ and VrPPase:P_i_ complexes have been deposited in the Protein Data Bank, www.rcsb.org (PDB IDs 5LZQ, 5LZR and 5GPJ). All other data that support the findings in this study are available from the corresponding authors upon reasonable request.

## Additional information

**How to cite this article:** Li, K.-M. *et al*. Membrane pyrophosphatases from *Thermotoga maritima* and *Vigna radiata* suggest a conserved coupling mechanism. *Nat. Commun.* **7,** 13596 doi: 10.1038/ncomms13596 (2016).

**Publisher's note**: Springer Nature remains neutral with regard to jurisdictional claims in published maps and institutional affiliations.

## Supplementary Material

Supplementary Information Supplementary Figures 1-9, Supplementary Tables 1-3 and Supplementary References

## Figures and Tables

**Figure 1:**
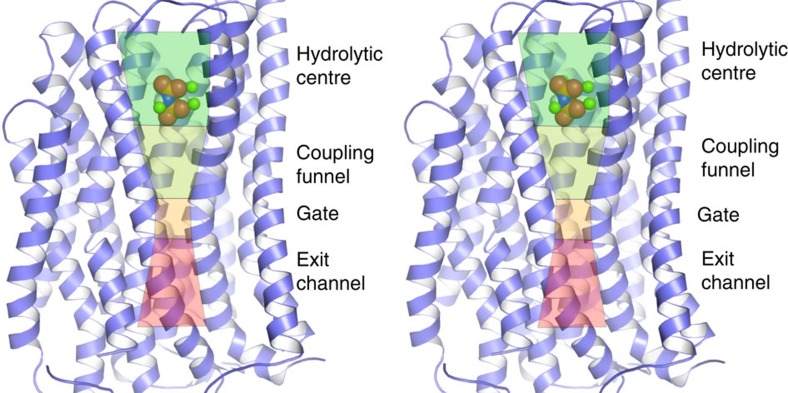
Stereo view of the vertical channel composition of M-PPases. Structural overview of an M-PPase:IDP complex, showing the various sub-structures in the active site: the hydrolytic centre, coupling funnel, ion gate and exit channel. Helices 5, 9, 10 and 15 have been removed for clarity.

**Table 1:** Structures and states of M-PPases.

**Protein**	**Resolution (Å)**	**No. chains/ASU**	**Active site contents**	**Closure of active site**	Position of TMH12*
TmPPase:Ca:Mg†	2.6	2	CaMg	Open: 3,400 Å^3^	0
TmPPase:IDP§	3.5	2	Mg_5_IDP (Na^+^ at ion gate)	Closed: 1,600 Å^3^	−2.7 Å
TmPPase:P_i2_†	4.0	2	Mg_4_P_i2_	ND||	−2.2 Å
TmPPase:WO_4_§	4.0	2	MgWO_4_	ND||	−1.0 Å
VrPPase:IDP‡	2.6	2	KMg_5_IDP	Closed: 1,600 Å^3^	−2.2 Å
VrPPase:P_i_§	3.5	4	Mg_2_P_i_	Open: 2,800 Å^3^	−0.7 Å

ASU, asymmetric unit; ND, not determined.

^*^Position relative to that in TmPPase:Ca:Mg based upon K^12.50^ C*α*, where a negative number represents a downwards motion.

^†^Structures from Kellosalo *et al*.^11^

^‡^Structure from Lin *et al*.^12^

^§^Structures presented in this paper.

^||^ND because the loops are missing, meaning that the active site volume can not be meaningfully compared. ASU, asymmetric unit.

**Table 2:** Data collection and refinement statistics.

**Crystal**	**TmPPase:IDP complex**	**TmPPase:WO**_**4**_	**VrPPase:P**_**i**_ **complex**
*Data collection*			
Space group	*P*2_1_2_1_2_1_	*P*2_1_	*C*2
Cell dimensions			
*a, b, c* (Å)	106.4, 106.8, 161.9	83.7, 108.9, 105.8	225.7, 81.6, 264.8
*β* (°)	90	108.8	92.9
Source	DLS—i04	ESRF ID23-1	NSRRC—BL15A1
Wavelength	0.9795	1.214	1.000
Resolution (Å)*	30–3.49 (3.70–3.49)	25–4.0 (4.1–4.0)	30.0–3.50 (3.62–3.50)
*R*_merge_ (%)*†	9.3 (140.8)	5.5 (42.6)	16.8 (117.5)
*I*/σ*	15.83 (1.35)	14.3 (3.65)	7.5 (1.11)
Completeness (%)*	99.5 (98.6)	98.8 (92.8)	92.2 (85.1)
Redundancy*	8.8 (8.6)	3.8 (3.4)	3.5 (3.5)
			
*Refinement*			
Resolution (Å)	3.5	4.0	3.5
No. of reflections	23,904	29,597	54,540
*R*_work_(%)/*R*_free_(%)	23.9/26.8	23.2/27.9	22.6/30.4
No. of atoms	10,443	9,808	21,413
Protein	10,389 (1,450 residues)	9,794 (1,383 residues)	21,385 (2,914 residues)
IDP, P_i_ or WO_4_	36 (2 IDP)	10 (2 WO_4_)	20 (4 P_i_)
Mg^2+^	10	2	8
Na^+^	2	–	–
No. of chains/ASU	2	2	4
B-factors (Å^2^)			
All atoms	139	134.9	119.6
Protein	138.9	133.0	119.5
IDP, P_i_ or WO_4_	167.8 (IDP)	198.5	159.4 (P_i_)
Mg	134.9	151.6	107.9
Na	112.7	–	–
r.m.s.d.			
Bond length (Å)	0.005	0.004	0.01
Bond angle (°)	1.06	1.19	1.48

^*^Values in the parenthesis are for the highest resolution shell.

^†^*R*_merge_=Σ|*I*−<*I*>|/Σ*I*, where *I*=observed intensity, and <*I*>=average intensity from multiple observation of symmetry related reflections.
