# Supplementary material for: Membrane pyrophosphatases from Thermotoga maritima and Vigna radiata suggest a conserved coupling mechanism
Source: Nat Commun. 2016 Dec 6;7:13596. doi: 10.1038/ncomms13596 (PMC5150537; doi:10.1038/ncomms13596)
Supplement: Supplementary Information — Supplementary Figures 1-9, Supplementary Tables 1-3 and Supplementary References [file ncomms13596-s1.pdf]

Supplementary Information

Supplementary figures

a

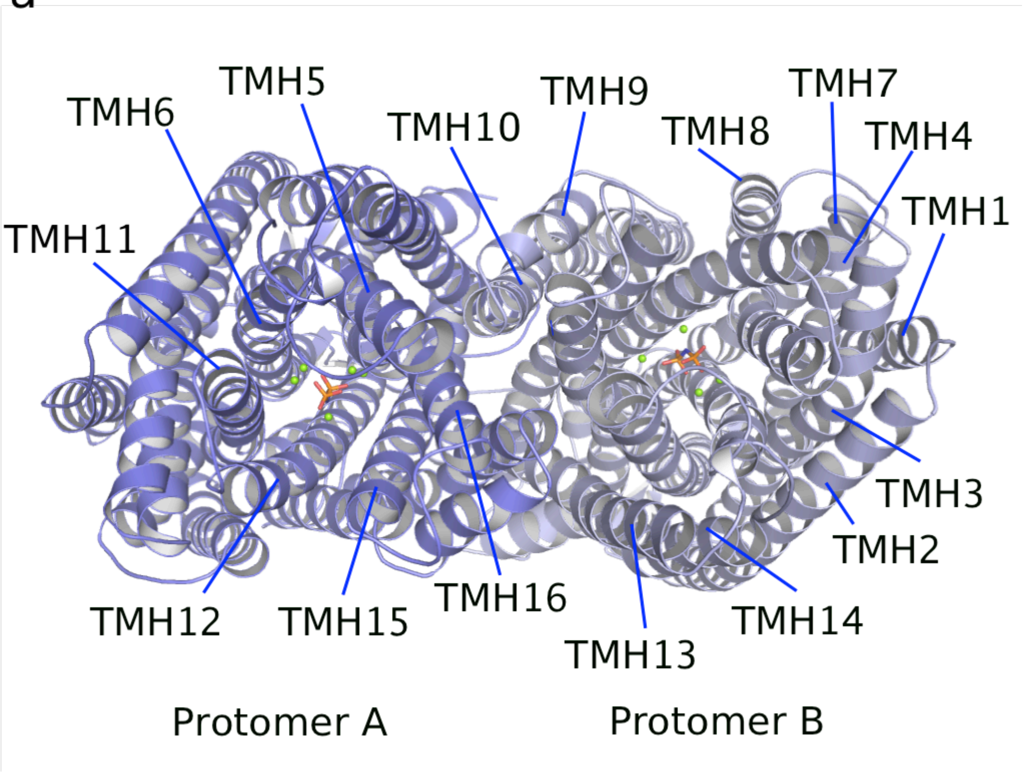

b

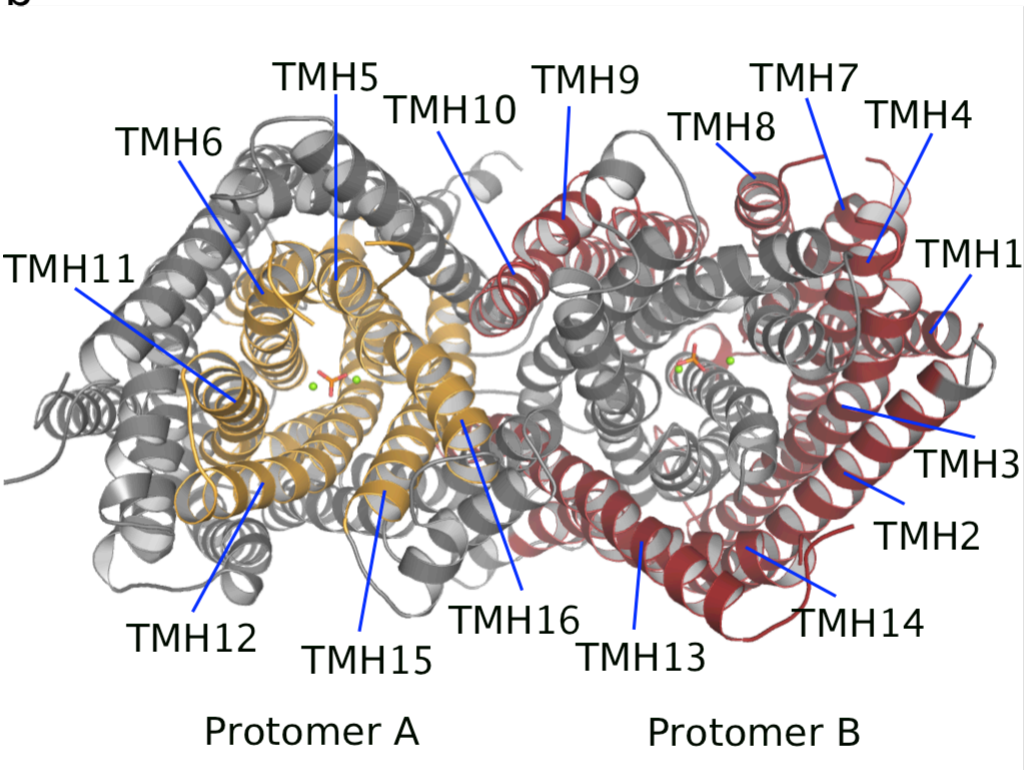

Supplementary Figure 1. Overviews of both TmPPase:IDP and VrPPase:P_i. viewed from above the cytoplasmic side above the hydrolytic center. (**a**) Overview of the TmPPase:IDP dimer, emphasizing how the two protomers form a dimer. The helices in protomer A are in blue and the ones in protomer B in gray. The IDP and Mg^{2+} complex for each protomer are shown in the hydrolytic center and are colored orange and green respectively. (**b**) Overview of the VrPPase:P_i dimer, highlighting the inner- (gold) and outer-ring (red) helical sub-structures of protomers A and B, respectively.

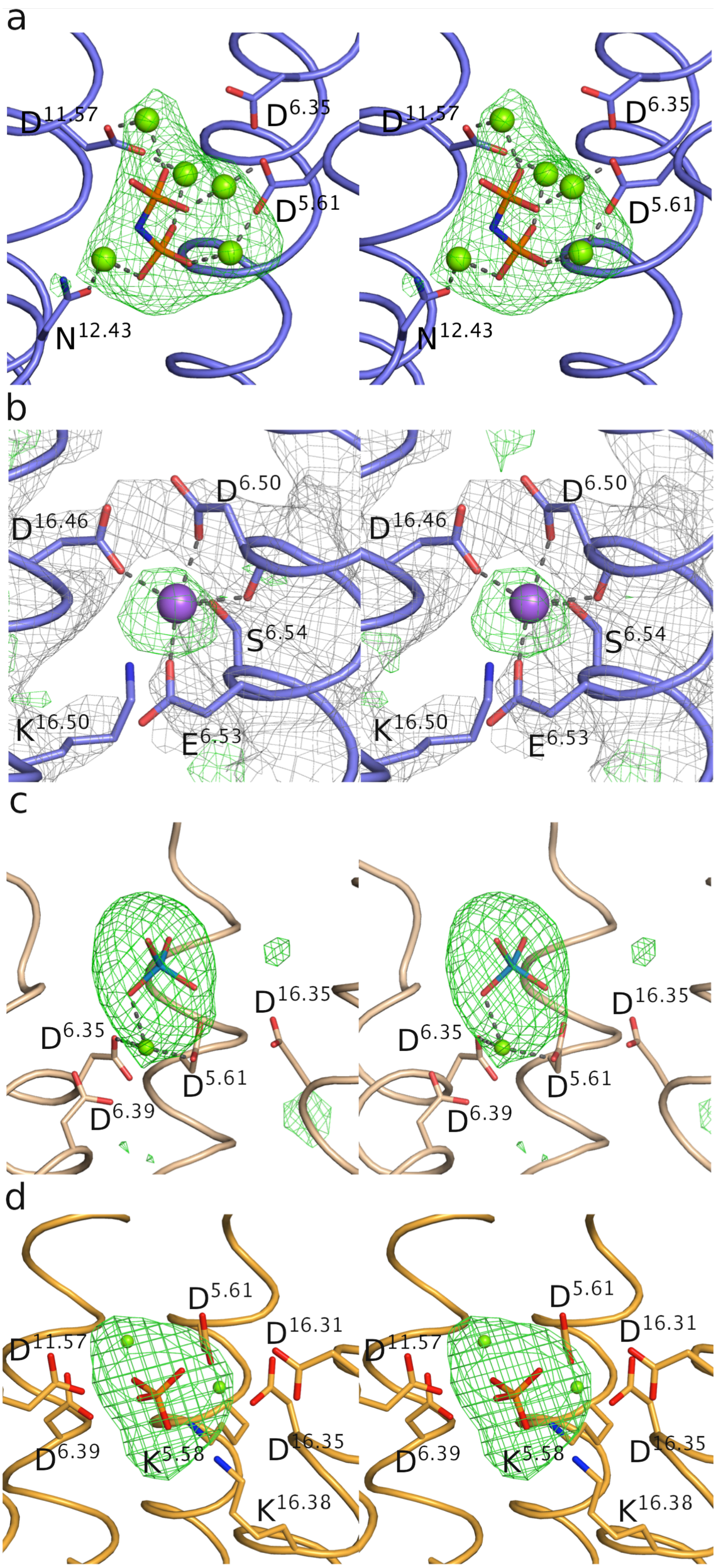

a
D11.57
D6.35
D5.61
N12.43
b
D6.50
D16.46
S6.54
K16.50
E6.53
c
D16.35
D6.35
D5.61
D6.39
d
D5.61
D16.31
D11.57
D6.39
K5.58
D16.35
K16.38

Supplementary Figure 2. Stereo views of F_o-F_c density of the hydrolytic centers and ion gate of TmPPase:IDP (blue), TmPPase:WO_4 (beige) and VrPPase:P_i (gold). F_o-F_c density (3 σ) is shown in fishnet for (**a**) the Mg:IDP complex of TmPPase in the hydrolytic center, (**b**) the bound Na^+ at the ion gate of TmPPase:IDP, (**c**) the Mg:WO_4 complex in the hydrolytic center of TmPPase:WO_4 and (**d**) the Mg:P_i complex in the hydrolytic center of VrPPase:P_i (at 6 σ). All of this is unbiased difference density calculated from the model before the ligands in question were added. $2F_o$-F_c density (1.5 σ) is also shown in (**c**) at a 1.6 Å radius for neighboring residues. IDP and P_i are shown in orange, Mg^{2+} are shown in green, Na^+ is shown in purple and WO_4^{2-} is shown in light blue and red.

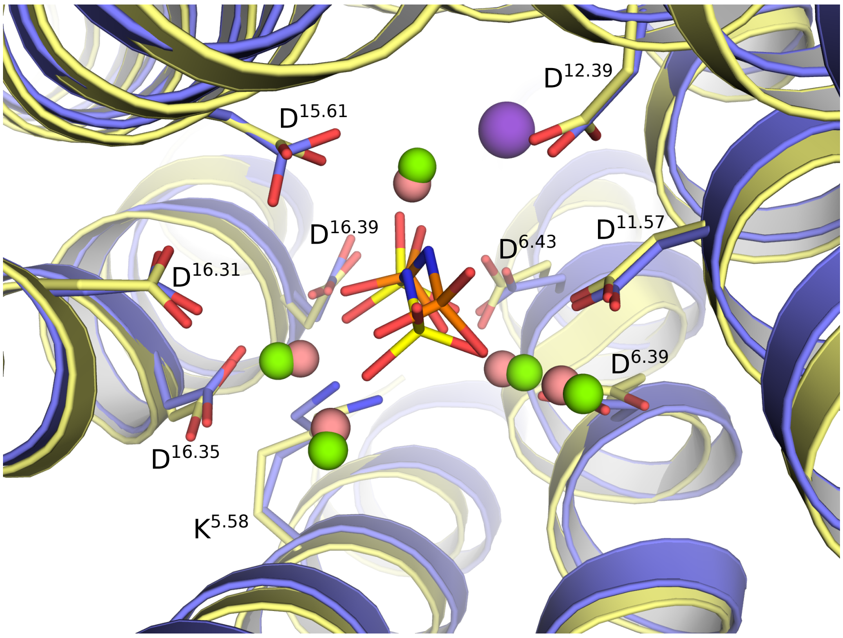

Supplementary Figure 3. The hydrolytic centers of TmPPase:IDP (blue) and VrPPase:IDP (yellow) structures, showing the similarities in how the Mg_4IDP complex binds to both proteins and of the active site. TmPPase:IDP: Ribbon backbone in blue with IDP in orange with and Mg^{2+} in green; VrPPase:IDP: Ribbon backbone in yellow, with IDP in yellow, Mg^{2+} in salmon and K^+ shown in purple. Coordinating residues are shown as sticks in atom colour, with the carbon atoms colored as the structures from which they come.

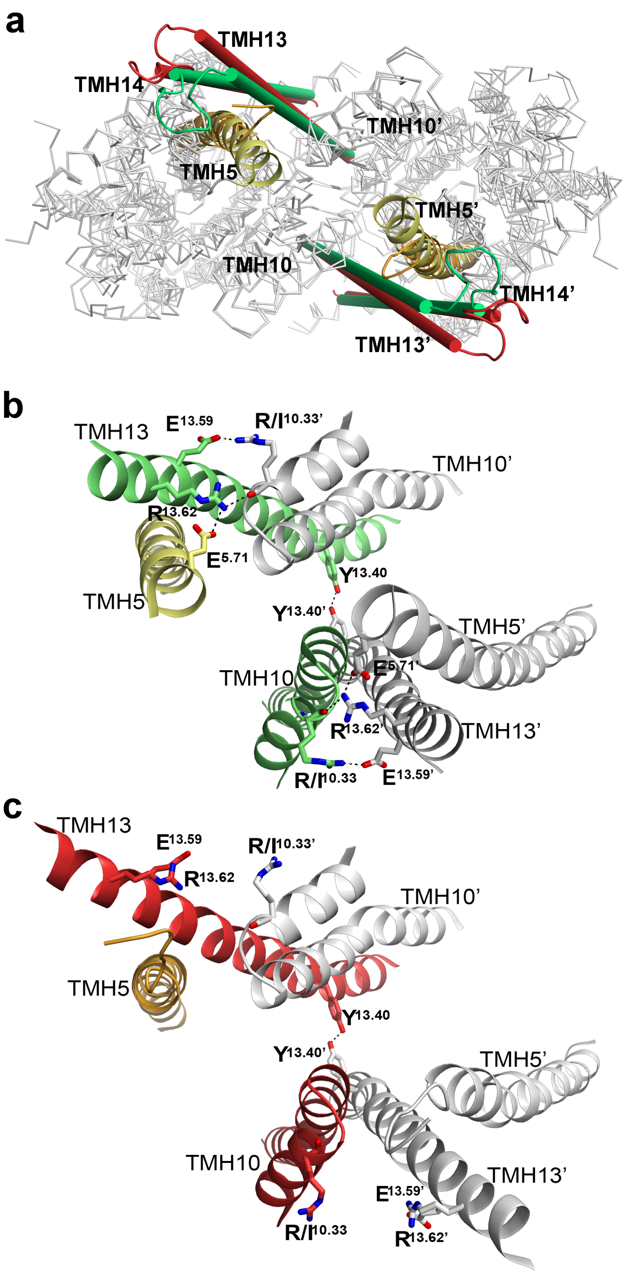

Supplementary Figure 4. Conformational change of TMHs 5, 13 and 14 in VrPPase. (**a**) Superimposition of the VrPPase:P_i (red and gold, this study) structure on the VrPPase:IDP complex structure (green and yellow). The overall structures of VrPPase are drawn as ribbon and colored in gray, but the TMHs 5-6 and TMHs 13-14 are drawn as ribbons and cylinders, respectively. The interactions in the IDP-bound (**b**) and P_i-bound (**c**) VrPPase complexes are shown, including the through $E^{5.71}$-$R^{13.62}$-$R^{10.33'}$ and the $E^{13.59}$- $R^{10.33'}$ interactions.

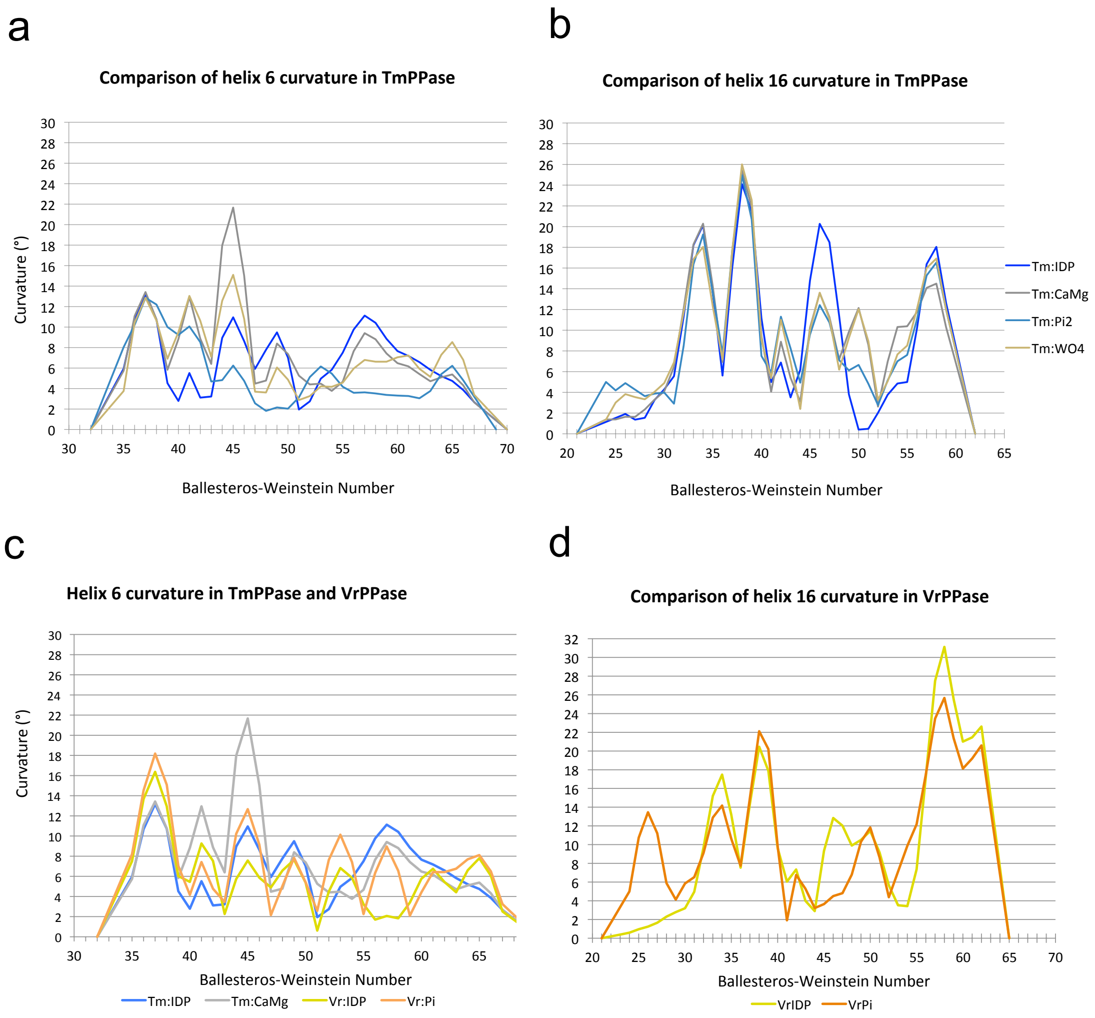

Supplementary Figure 5. Helix curvature between different catalytic states of M-PPases. The change in helix curvature is shown for (**a**) helix 6 between the 4 main states of TmPPase (**b**) helix 16 between the four main states of TmPPase, (**c**) helix 6 between different states of both TmPPase and VrPPase and (**d**) helix 16 between both states of VrPPase.

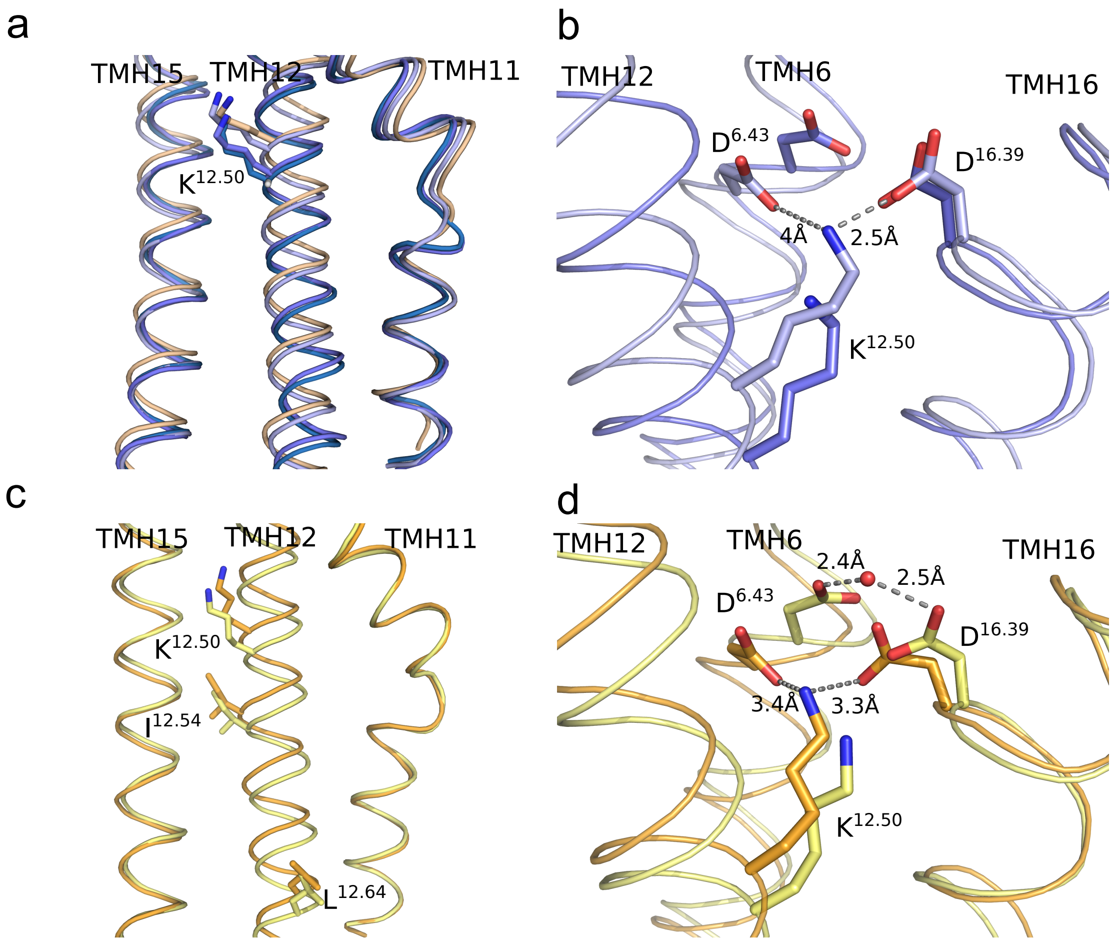

Supplementary Figure 6. Movement of TMH12. (**a**) Movement of TMH12 across the 4 states of TmPPase: Substrate-analogue-bound (TmPPase:IDP, blue), product-bound (TmPPase:P_{i2}, sky blue), resting-state, (TmPPase:Ca:Mg, light blue) and tungstate-bound (TmPPase:WO_4, beige) including positions of $K^{12.50}$ across these states; and (**b**) the breakage of the ion pair between $D^{6.43}$ and $K^{12.50}$ upon substrate binding in TmPPase. (**c**) TMH12 movement between the 2 states of VrPPase: substrate-analogue-bound (VrPPase:IDP, yellow) and product-bound (VrPPase:P_i, gold) including the positions of $K^{12.50}$, $I^{12.54}$ and $L^{12.64}$ across these states. (d) The interactions of $D^{6.43}$, $K^{12.50}$, $D^{16.39}$ and the nucleophilic water (red) between the two states of VrPPase.

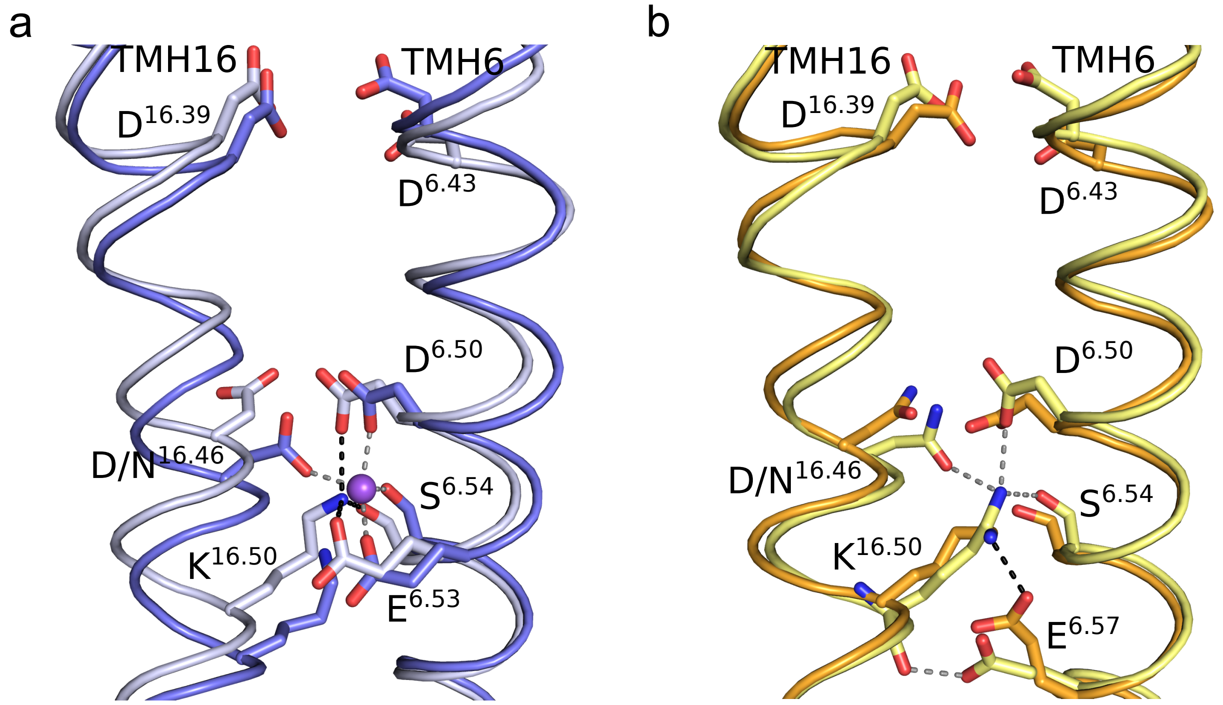

Supplementary Figure 7. Movement of TMHs 6 and 16 in comparisons of (a) TmPPase:IDP (blue) and TmPPase:Ca:Mg (light blue) and (b) VrPPase:IDP (yellow) and VrPPase:P$_i$ (gold). The images show the changes in the positions of $D^{6.43}$ and $D^{16.39}$ and (**a**) the bound Na^+ (purple) in TmPPase:IDP and its coordination bonds (grey dashes), the salt-bridge network in TmPPase:Ca:Mg (black dashes) and (**b**) the bonding network in VrPPase:IDP (grey dashes) and VrPPase:P$_i$ (black dashes). (**a**) shows the change in position from $K^{16.50}$ bound to $D^{6.50}$ and $D^{6.53}$ in TmPPase:Ca:Mg (light blue) to disordered and replaced by the Na^+ in TmPPase:IDP (blue).

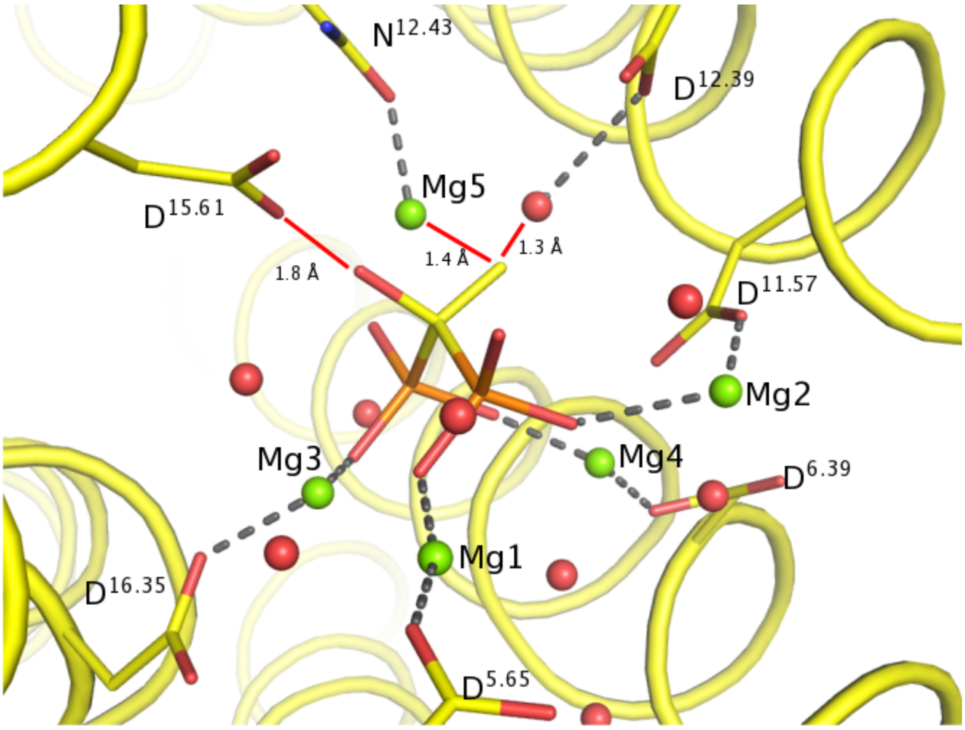

Supplementary Figure 8. Docking of etidronate into the active site of VrPPase.

Etidronate was docked so that the two phosphate moieties were positioned on the phosphate moieties in IDP in both possible orientations. There were steric clashes in both orientations. In the one shown, possibly the less disfavoured one, the extra OH group clashes with the neighbouring aspartate residue $D^{15.61}$ (VrPPase D691) (2Å) and with the fifth magnesium ion (Mg5) (2Å), while the $–CH_3$ group clashes with Mg. There is no doubt that this would prevent, or at least greatly interfere with, closing of the active-site loops

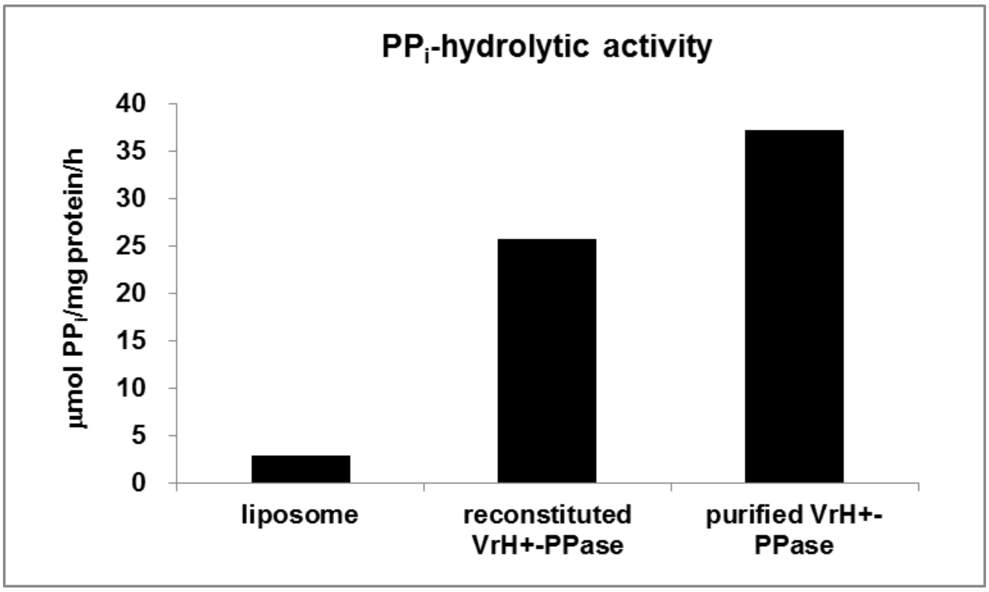

Supplementary Figure 9. PP_i-hydrolytic activity. Hydrolytic activities of the purified and reconstituted *Vr*H^+-PPase protein samples alongside control liposomes without protein. This assay was carried out using the molybdate coloring method[1].

Supplementary Table 1. M-PPase Ballesteros and Weinstein numbering[2] (adapted from [3]).

TMH	Residue in Tm/Vr-PPase	B & W Signifier*	% identity†
1	F17/F25	1.50	38.1
2	K55/K94	2.50	27.6
3	S87/S153	3.50	99.5
4	G130/G194	4.50	92.9
5	R191/R242	5.50	86.4
6	D243/D294	6.50	99.7
7	G297/G334	7.50	46.3
8	L321/L361	8.50	75.8
9	G369/G411	9.50	91.1
10	S416/S458	10.50	84.3
11	D458/D500	11.50	98.1
12	K499/K541	12.50	99.7
13	V566/V597	13.50	99.4
14	M611/M642	14.50	79.1
15	A649/A680	15.50	94.0
16	K707/K742	16.50	94.5

* In each TMH, residues towards the N-terminus from the amino-acid $X^{x.50}$ have B & W signifiers $X^{x.49}$, $X^{x.48}$, $X^{x.47}$ etc., while residues towards the C-terminus have signifiers $X^{x.51}$, $X^{x.52}$, $X^{x.53}$ etc.,

† Sequence identity of this residue in a multiple sequence alignment of all of the M-PPases in the UniProt database.

Supplementary Table 2: Hydrogen bond patterns for helices 6 and 16 within different states of TmPPase and VrPPase

Residue from	Residue to (Helical Geometry)			
	**TmPPase:IDP**	**TmPPase:CaMg**	**VrPPase:IDP**	**VrPPase:P$_i$**
**$N^{6.40}$**	X	$V^{6.37}$ (3_{10})	$V^{6.37}$ (3_{10})	X
**$V^{6.41}$**	$V^{6.37}$ (α)	$V^{6.37}$ (α)	$G^{6.38}$ (3_{10})	$V^{6.37}$ (α)
**$G^{6.42}$**	$G^{6.38}$ (α)	$G^{6.38}$ (α)	$G^{6.38}$ (α)	$G^{6.38}$ (α)
**$D^{6.43}$**	X	$D^{6.39}$ (α)	X	$D^{6.39}$ (α)
**$V/I^{6.44}$**	$D^{6.39}$ (π)	$N^{6.40}$ (α)	$D^{6.39}$ (π)	$N^{6.40}$ (α)
**$A^{6.45}$**	$N^{6.40}$ (π)	$N^{6.40}$ (π)	$N^{6.40}$ (π)	$V^{6.41}$ (α)
**$G^{6.46}$**	$G^{6.42}$ (α)	$V^{6.41}$ (π)	$G^{6.42}$ (α)	$V^{6.41}$ (π)
**$L/M^{6.47}$**	$I^{6.44}$ (3_{10})	$G^{6.42}$ (π)	$G^{6.42}$ (π)	$G^{6.42}$ (α)
**$V/S^{16.41}$**	$P^{16.36}$ (π)	$P^{16.36}$ (π)	$P^{16.36}$ (π)	$P^{16.36}$ (π)
**$G^{16.42}$**	$L^{16.37}$ (π)	$L^{16.37}$ (π)	$L^{16.37}$ (π)	$L^{16.37}$ (π)
**$P^{16.43}$**	X	X	X	X
**$S^{16.44}$**	$V/S^{16.41}$ (3_{10})	$T^{16.40}$ (α)	$V/S^{16.41}$ (3_{10})	$T^{16.40}$ (α)
**$L^{16.45}$**	$G^{16.42}$ (3_{10})	$V/S^{16.41}$ (α)	$V/S^{16.41}$ (α)	$G^{16.42}$ (3_{10})
**$D/N^{16.46}$**	X	X	X	X
**$I^{16.47}$**	$S^{16.44}$ (3_{10})	$S^{16.44}$ (3_{10})	$S^{16.44}$ (3_{10})	$S^{16.44}$ (3_{10})
**$L^{16.48}$**	$S^{16.44}$ (α)	$S^{16.44}$ (α)	$S^{16.44}$ (α)	$S^{16.44}$ (α)

X refers to instances where no interactions are made. The backbone (a, p, 3_{10}) hydrogen bond was considered to be formed if N---O=C bend angle $\geq 145^o$ and N---O distance $\leq$ 4Å.

Supplementary Table 3. Mutagenesis of residues in the protomer-protomer interface[4]

Mutant in ScPPase	Hydrolysis activity*	H^+ pump activity*	Coupling efficiency*	Residue in ScPPase**	Corresponding Residue in Vr/Tm-PPase	Interacting Residue† in Vr/Tm-PPase
No hydrolytic activity	0	0	0	$V^{10.40}A$	$V/I^{10.40}$	$I/V^{16.33}$
	0	0	0	$L^{10.48}P$	$Y/M^{10.48}$	$M/I^{13.47}$
	0	0	0	$L^{13.41}Q$	$W/Y^{13.41}$	$I^{10.52}/K^{10.49}$
Loose-coupling	~40%	~10%	25%	$I^{10.44}T$	$L/L^{10.44}$	$M/I^{13.47}$
	25%	~5%	~15%	$V^{10.52}I$	$I/F^{10.52}$	$W^{13.41}/I^{13.38}$
	120%	~30%	~30%	$F^{13.40}L$	$Y/Y^{13.40}$	$Y/M^{10.48}$

* Activity and efficiency calculated relative to that of wild-type *Streptomyces coelicolor* PPase (ScPPase)

** The residue substituted with other amino acid in *Streptomyces coelicolor* PPase (ScPPase) and its corresponding residue in VrPPase and TmPPase.

† The dimer interacting residue partner in VrPPase and TmPPase observed in the crystal structures.

Supplementary References

1. Liu, T.H., *et al.* The proximity between C-termini of dimeric vacuolar H^+-pyrophosphatase determined using atomic force microscopy and a gold nanoparticle technique. *FEBS J.* **276**, 4381-4394 (2009).

2. Ballesteros, J.A. & Weinstein, H. Integrated methods for the construction of three-dimensional models and computational probing of structure-function relations in G protein-coupled receptors. *Methods Neurosci.* **25**, 366-428 (1995).

3. Tsai, J.Y., Kellosalo, J., Sun, Y.J. & Goldman, A. Proton/sodium pumping pyrophosphatases: the last of the primary ion pumps. *Curr. Opin. Struct. Biol.* **27**, 38-47 (2014).

4. Hirono, M., Nakanishi, Y. & Maeshima, M. Essential amino acid residues in the central transmembrane domains and loops for energy coupling of *Streptomyces coelicolor* A3(2) H^+-pyrophosphatase. *Biochim. Biophys. Acta* **1767**, 930-939 (2007).
